# Statistical learning in songbirds: from self-tutoring to song culture

**DOI:** 10.1098/rstb.2016.0053

**Published:** 2017-01-05

**Authors:** Olga Fehér, Iva Ljubičić, Kenta Suzuki, Kazuo Okanoya, Ofer Tchernichovski

**Affiliations:** 1 School of Philosophy, Psychology and Language Sciences, University of Edinburgh, 3 Charles Street, Edinburgh EH8 9AD, UK; 2 Psychology Department, Hunter College, 695 Park Avenue, New York, NY 10065, USA; 3 Biology Department, The Graduate Center, CUNY, 365 Fifth Avenue, New York, NY 10016, USA; 4 Faculty of Health Sciences, Nihon Institute of Medical Science, 1276 Shimogawara, Moroyama-machi, Iruma-gun, Saitama 350-0435, Japan; 5 Department of Life Sciences, University of Tokyo, 3-8-1 Komaba, Meguro-ku, Tokyo 153-8902, Japan; 6 Psychology Department, The Graduate Center, CUNY, 365 Fifth Avenue, New York, NY 10016, USA

**Keywords:** birdsong, development, isolate song, statistical learning, categorical signal, song culture

## Abstract

At the onset of vocal development, both songbirds and humans produce variable vocal babbling with broadly distributed acoustic features. Over development, these vocalizations differentiate into the well-defined, categorical signals that characterize adult vocal behaviour. A broadly distributed signal is ideal for vocal exploration, that is, for matching vocal production to the statistics of the sensory input. The developmental transition to categorical signals is a gradual process during which the vocal output becomes differentiated and stable. But does it require categorical input? We trained juvenile zebra finches with playbacks of their own developing song, produced just a few moments earlier, updated continuously over development. Although the vocalizations of these self-tutored (ST) birds were initially broadly distributed, birds quickly developed categorical signals, as fast as birds that were trained with a categorical, adult song template. By contrast, siblings of those birds that received no training (isolates) developed phonological categories much more slowly and never reached the same level of category differentiation as their ST brothers. Therefore, instead of simply mirroring the statistical properties of their sensory input, songbirds actively transform it into distinct categories. We suggest that the early self-generation of phonological categories facilitates the establishment of vocal culture by making the song easier to transmit at the micro level, while promoting stability of shared vocabulary at the group level over generations.

This article is part of the themed issue ‘New frontiers for statistical learning in the cognitive sciences’.

## 1. Introduction

The ability to recognize and internalize recurring patterns in the environment provides animals with many ecological advantages by facilitating learning and aiding generalization to new stimuli and allowing them to predict upcoming events. Rule or pattern learning is ubiquitous in the animal kingdom in every sensory domain, and it may occur via statistical learning. Statistical learning, when patterns are identified by the extraction of statistical information inherent in sensory stimuli, has been demonstrated in humans in all sensory modalities and in many different types of learning tasks [1–3]. A major area of research concerns its role in language acquisition. Humans are able to extract statistical regularities from their auditory environment from a very young age. Pre-linguistic infants, for instance, can acquire several types of statistical information from linguistic stimuli: conditional relations [4], distributional frequencies [5], structural information [6] and cue-based statistics (when a perceptible attribute of the input is correlated with an unobservable attribute). These types of statistical learning all play a role in language acquisition [7], but it is unclear whether the underlying learning mechanisms are shared with other animals. Here, we examine one type of statistical learning: the extraction of categories from auditory input during vocal learning in songbirds. This type of statistical learning is of particular interest because, at least superficially, it appears similar to the process in which human infants acquire phonetic categories during early speech development.

Statistical learning abilities have been demonstrated in primates [8,9], songbirds [10–12] and rodents [13]. Statistical learning in songbirds have been investigated using a variety of tasks involving phonological and syntax perception and artificial grammar learning [11,14,15]. In natural song development, songbirds have been shown to rely on statistical information in sequential learning [14,16]. At the neuronal level, recordings in the auditory forebrain of awake birds reveals sensitivity to statistical regularities, even to non-adjacent patterns [17]. However, there are differences between species in terms of generalization abilities and preferred learning strategies. For instance, in an artificial grammar learning task, zebra finches (whose songs are highly stereotyped) were found to generalize based on positional relationships, while budgerigars (a parrot species that produces long and variable songs) were able to extract the underlying abstract rules between items [12]. One of the central questions in statistical learning research is how species- or domain-general the underlying learning mechanisms are [18], so it is of particular interest to study statistical learning mechanisms that support a more general ability to vocally learn in order to gain a deeper understanding about the specializations that led to the emergence of human language.

Vocal learning, the ability to modify vocal output based on auditory input, is rare in nature, only present in a few animal taxa, and it is best studied in songbirds. Songbirds provide a useful animal model to investigate the biological foundations of human linguistic behaviour, because there are numerous parallels in the behavioural, neural and genetic mechanisms between birdsong and spoken language [19–21]. Vocal learners, such as humans and songbirds, acquire complex vocalizations from adult individuals during development. In both songbirds and humans, vocal development is constrained by biases that guide acquisition and shape the structure of the communication system (e.g. [22,23]). Moreover, songbirds exhibit categorical perception of note types [24], which is dependent on context [25], and the category boundaries differ among subpopulations within the same species [26] just as human languages differ in their phoneme inventories. In humans, statistical learning is involved in the development of the perception of phonetic categories [5,27], but we do not know how statistical learning contributes to the development of phonetic categories in birdsong.

Studying vocal development in songbirds in the laboratory gives us full control over their sensory input while recording their entire vocal ontogeny. Here, we use a songbird, the zebra finch (*Taeniopygia guttata*), to investigate how distributional information in the vocal input affects the development of phonetic categories. We compared three conditions: no input, namely, complete social and acoustic isolation (ISO group), exposure to song playbacks that approximate the natural, categorical song input (wild-type, WT group) and self-input: exposing birds to playbacks of their own developing song, which is initially non-categorical (self-tutored, ST group). The input to the ST group was constantly updated during development. Comparing these groups allowed us to measure the time-course of phonological category formation and to judge what statistical features of song structure are input-dependent and which ones can be internally generated.

**Figure 1.**
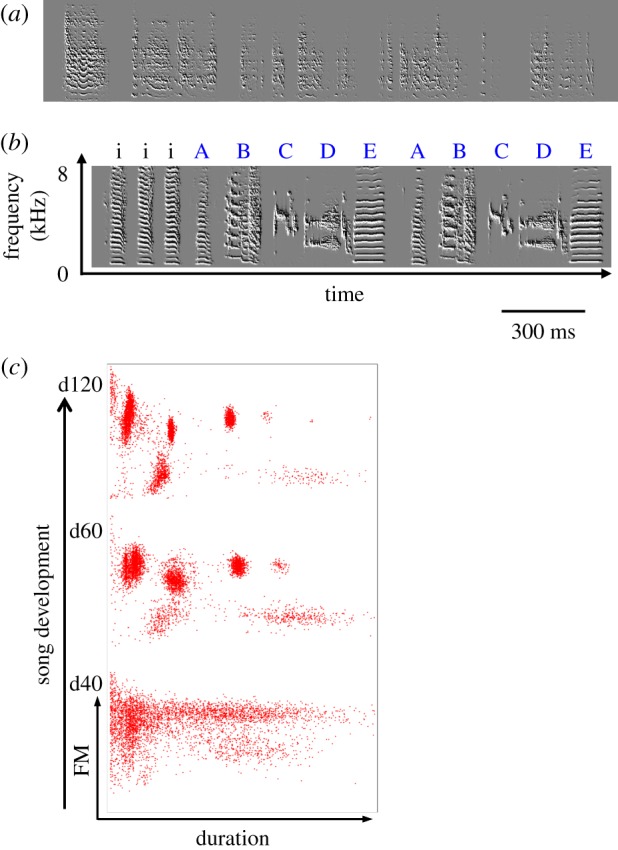
Transition from continuous to categorical signal over development. (*a*) Sonogram of a juvenile zebra finch (day 40) showing highly variable, undifferentiated syllables. (*b*) Adult song of the same zebra finch showing stable syllable types falling into distinct categories. Introductory syllables are denoted by i; letters stand for song syllables that are repeated in a fixed order (ABCDE…). (*c*) Development of syllable types (categorical signal) in a WT bird. Dots represent song syllables: mean frequency modulation (*y* axis) is plotted against syllable duration (*x* axis, both normalized). Bottom panel represents distribution of syllables at the subsong stage, top panel at adulthood.

The time-course of song development in zebra finches has been studied extensively: vocal development begins with babbling at about day 30 post-hatch and ends around day 90 (figure 1). Zebra finches sing intensely during this period, producing about one million song syllables. Their entire vocal development can be recorded and analysed, making it possible to track moment-to-moment changes that occur during development [28]. The early song, called subsong (figure 1*a*), is highly variable, and acoustic features such as syllable duration and pitch are broadly distributed with no apparent clusters (figure 1*c*, bottom panel). In most birds, the first developmental changes in song structure can be seen at about day 50, when the broad distribution of song features changes, and distinct clusters begin to form (figure 1*c*, middle panel). These clusters become increasingly dense and tight, until they appear as distinct syllable types in the mature zebra finch song (figure 1*c*, top panel). The song by this stage is a categorical signal, composed of syllable types organized into motif units produced in a fixed order (figure 1*b*). After song crystallizes, at about day 100, adult zebra finches retain their stereotyped song motif for the rest of their life.

In the absence of song tutoring, socially isolated zebra finches improvize an abnormal song, which typically lacks a stable motif and shows high acoustic variability across renditions compared with WT songs [29]. In a previous study, we showed that WT song features emerge de novo over a few generations when the song of an isolate bird is passed from bird to bird in iterated learning chains [30]. This suggests that zebra finches have learning biases that steer songs towards WT features even when WT song input is absent. But why is the ISO song abnormal to begin with? The forebrain (cortical) song system is composed of two functionally distinct anatomical pathways: a posterior premotor pathway responsible for adult song production and an anterior forebrain (basal ganglia) pathway, which is involved in early song production (vocal babbling) and in song learning [31–35]. The premotor song system receives auditory input and responds strongly to song playbacks, most strongly to the bird's own song [36,37]. This sensory activation of the song system is crucial for song learning [38]. However, during singing, the motor song system becomes momentarily ‘deaf’ [39], probably in order to avoid simultaneous sensory and motor activation of the same neurons. Therefore, although the auditory feedback of the bird's own song affects song development even in ISOs [40], it seems unlikely that they can use their own vocalizations as a sensory template for song learning. In this study, we bypass the biological barrier that prevents ISOs from forming a song template by ‘tricking’ young birds into imitating delayed playbacks of their own developing song.

**Figure 2.**
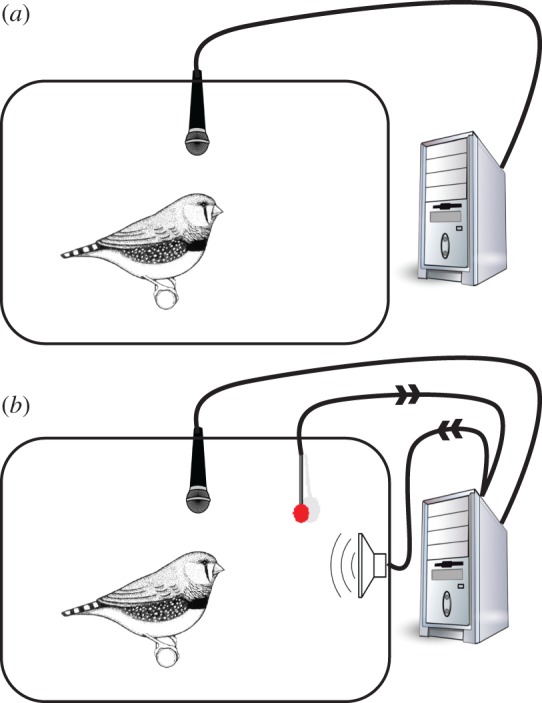
Self-tutoring in zebra finches. (*a*) ISO siblings are raised in social isolation without any song input; their vocalizations are recorded continuously. (*b*) ST birds are also socially isolated and recorded during the entire song development, but in addition, they learn to peck on a key (red button) that induces playback of a song randomly selected from their own recent vocalizations, repeated recursively over development.

Training zebra finches with self-input closes a very simple feedback-loop: it may induce vocal learning without any external input, through iterated learning occurring many thousands of times over development (figure 2). This process is similar to inter-individual iterated learning, and it may reveal internal biases in a similar fashion but in this case over cycles of self-imitation [41]. The principal question here is whether self-iterated learning is sufficient to steer the vocal output towards WT song features, and crucially, to evaluate if birds can efficiently generate phonetic categories in the absence of external categorical input. In other words, our method allows us to test if the formation of phonetic categories is contingent on the availability of categorical input.

## 2. Material and methods

### (a) Animal care

Twelve brother pairs were selected from the Hunter College breeding colony for the experiment. All birds were raised by their parents in a dedicated cage in a colony room until day 7 post-hatching. The father was then removed, and the cage was taken to a nursery area housing mothers (who do not sing) and chicks only. Birds were raised by their mother, and at day 30, at the onset of song development, the male siblings were separated from the mother and placed singly in sound-attenuated recording boxes, where they remained until adulthood (day 120). One bird of each sibling pair was raised in social and acoustic isolation (isolate, ISO group), while the other was trained on its own vocal output (self-tutored, ST group). We used brothers to minimize the influence of genetic variation in pairwise statistical comparisons.

### (b) Experimental groups

#### (i) Self-tutored group (ST, 12 birds)

One bird from each of our 12 sibling pairs was randomly selected to undergo training using operant song playback, as in [28]. Training began 2–4 days after placement into the sound boxes, when the birds were 33–35 days old, to allow familiarization with the new environment. Birds were trained on their own developing song (figure 2). To do this, we used Sound Analysis Pro 2011 (SAP2011) [42] to automatically record the entire vocal output of each bird, continuously over development. We developed an extension to SAP2011, which, on a separate thread, automatically identified song bouts produced by the bird, in nearly real-time. The software identified as putative song any vocalization (song bout) that lasted more than 1 s containing silence periods of no more than 100 ms, and selected those that included frequency-modulated sounds, to exclude bouts of non-modulated calls. This procedure gave a false-positive error rate of up to 30%, though it was typically much lower. False-positive song bouts included mostly bouts of broadband cage noise. The software saved the 10 most recent putative song bouts to a song library folder, for each bird separately. The content of the song library folder was updated every 20 min for each bird. Given that juvenile zebra finches typically produce thousands of song bouts every day, turnover rate of songs in each library was less than 1 h, except during night sleep. The sound box contained a switch with a key—each time the bird pecked on the key, one of the 10 recent songs was randomly played through a speaker placed behind a plastic bird model in the cage. Most birds pecked on the keys to hear songs hundreds of times every day, although there was considerable individual variation (mean number of key pecks = 463, s.d. = 236). This training continued until the bird was 120 days old, at which time we discontinued the experiment and moved the bird into a colony room.

#### (ii) Isolate group (ISO, 12 birds)

Brothers of each bird in the ST group were isolated from day 30 to day 120, as the ST group, but did not receive any training with song playbacks. With the exception of lacking key switches, their cages were identical to those of the ST birds, including a plastic bird model (which did not produce any sound). Vocalizations produced by the ISO birds were recorded continuously.

#### (iii) WT training group (WT, 8 birds)

Similarly to the ST group, WT birds pecked on keys to activate playbacks of the tutor song, with the difference that the total amount of song exposure was limited to two daily sessions of 10 songs each. This was done in order to facilitate song imitation, because imitation success had been shown to be inversely related to model abundance [43]. Each WT bird was trained with a single randomly selected WT fully stereotyped adult song, as in [28]. All eight birds produced either partial or complete imitations of the WT song by day 105 when we terminated the experiments. We used their complete song development records, day 40–100 post-hatch, as a normal song learning (WT) control.

### (c) Data analysis

We analysed the vocal output of each bird throughout development. For each bird, we took all the vocalization data produced every 5th day, starting from day 40 post-hatch. Vocalizations were processed in the Batch Processing module of SAP2011, which automatically segments and calculates mean and variance values for several acoustic features and saves them in a MySQL database. We used six syllable features for analysis: syllable duration, mean amplitude, mean pitch, mean frequency modulation (the angular component of the frequency contour, a measure of how modulated song notes are), mean Wiener entropy (a measure of the uniformity of sound—higher values representing noisy sounds, very low values indicating pure tones) and mean goodness of pitch (an estimate of harmonic pitch periodicity—high values corresponding to harmonic stacks regardless of modulation). For a formal definition of the acoustic features used in this study, see http://soundanalysispro.com/manual-1/chapter-4-the-song-features-of-sap2/contents. Together, these features summarize much of the acoustic structure of each syllable. Matlab and R (v. 3.1.0) were used for further analysis of the acoustic features.

#### (i) K-nearest neighbour analysis

From each day (40, 45, 50, etc.), 5000 syllables were randomly sampled from all the song syllables produced. We then scaled the syllable features as in [43], and computed Euclidean distances for each pair of syllables across all features to obtain a 5000 × 5000 matrix of acoustic distances. In order to obtain a robust estimate of cluster density, we calculated the median of the 100 closest neighbours (which are likely to be at the centre of clusters) as our measure.

#### (ii) Statistical analysis

To test for differences in developmental trajectories between the ISO and ST groups, we used a linear mixed effects model in R using the lme4 package [44]. We compared the training conditions in a pairwise manner, resulting in three comparisons: ISO and ST, ST and WT and ISO and WT. We defined age as a numerical value and set the intercept to day 40, the first day that we included in our analysis. We defined a null model that included only developmental age as a fixed effect, and two training models that added a fixed effect for training condition or a fixed effect for training condition plus an age by training condition interaction. To account for the random effect of bird identity, all models included by-bird random intercepts for age. *P*-values for fixed effects were obtained via model comparison. To analyse the time-course of the divergence between ISO and ST birds, we performed *t*-tests at every time-point with false-discovery rate adjustment for multiple comparisons. Empirical cumulative distribution functions (ECDFs) were calculated for the final day of song production separately for each feature, and differences were quantified using Kolmogorov–Smirnov tests.

## 3. Results

**Figure 3.**
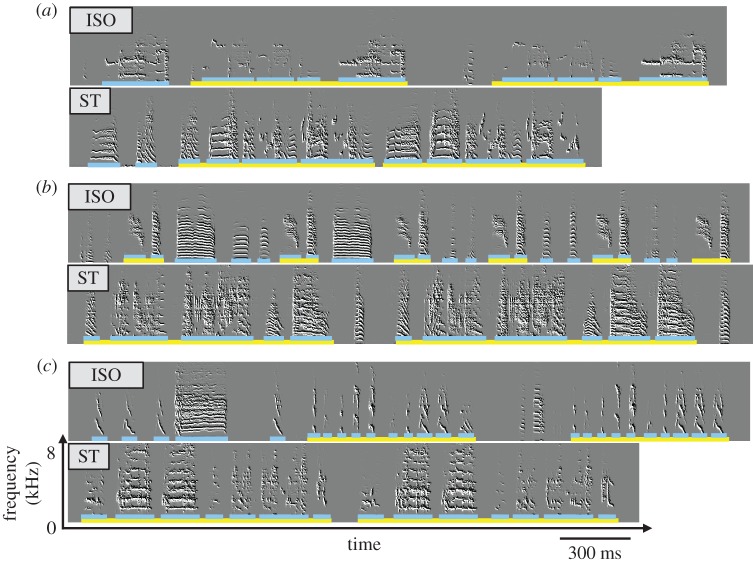
Adult songs of ISO and ST (ST) sibling pairs. Sonograms of three pairs are shown (*a–c*). Songs of the ISO brothers are above their ST siblings'. Song motifs are underlined in yellow, song syllables in blue. ST motifs are more stable (repeated in the same way every time), composed of more syllable types and are generally longer. ST syllable durations are similar to the WT durations; ISO durations tend to be too short or too long.

Birds that were trained with their own developing songs (ST), produced songs that were very different from those of their ISO brothers. Figure 3 shows example sonograms for three sibling pairs demonstrating that ST songs were both more structured and more stereotyped than ISO songs. The ST song motifs (underlined in yellow) tended to contain a larger variety of syllable types (underlined in blue) and were more stereotyped, and similar to WT zebra finch motifs. Most of the ST song syllables (figure 3*a,b*) contained multiple notes and high frequency modulation. By contrast, ISO syllables were often either very long with a non-modulated call-like structure (figure 3*b*), or very short clicks (figure 3*c*) that are rare in WT zebra finch songs. We did not observe any clicks in songs of ST birds.

**Figure 4.**
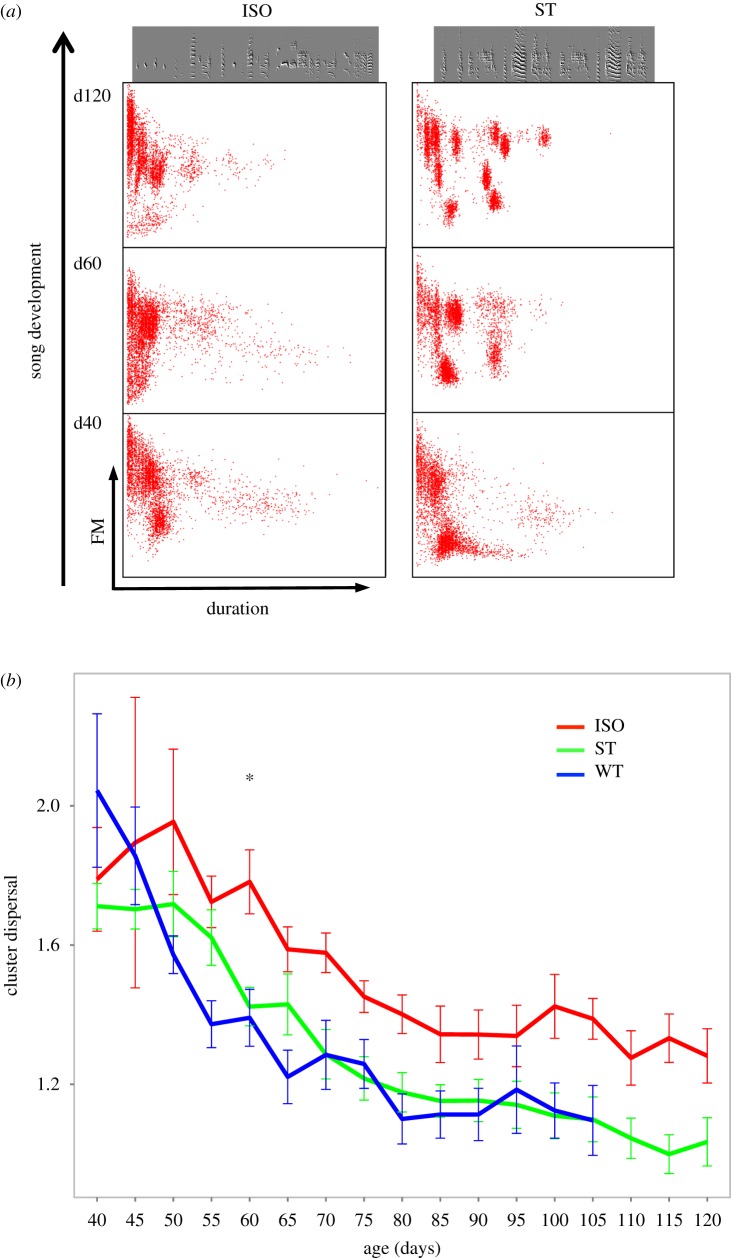
Syllables form tight clusters in WT and ST birds but not in ISOs. (*a*) Cluster formation in an ISO bird (left column), his ST brother (right column). Dots represent song syllables: mean frequency modulation is plotted against syllable duration (both normalized). The top panels represent adult songs, the bottom ones the beginning of development, and the middle row a stage in early development when ST birds have already begun to produce stable and tightly clustered syllables not present in ISOs. (*b*) Median Euclidean distance between neighbouring syllables. Over song ontogeny, syllables of the same type become more similar in every group, but this happens faster and earlier in self- and WT birds while the ISOs never from tight clusters.

We visualize the process of syllable type (category) formation and stabilization over development using scatter-plots of syllable features at different stages of song development [45]. Figure 4*a* presents an example of an ISO bird and his ST brother's development. For each syllable (red dot), duration is plotted against frequency modulation, and each panel shows 3000 song syllables produced on a particular day by one bird. At the beginning of song development, both birds produced unstructured and highly variable syllables that take up a large diffuse area in feature space without clear categories (lower panels). However, as development progressed (towards upper panels in figure 4*a*), the birds formed clusters of syllable types. By the end of development, as the songs were crystallized and repeated with low variability, the clusters become small and tight. In most ST birds, similarly to WT birds, clear clusters corresponding to categorical signals were present by day 60, but in ISO birds the syllables at this time were still diffuse, and even at the end of song development, the categories were not as clearly defined as in ST and WT birds.

To quantify the emergence of categories (clusters) over development, we calculated Euclidean distances between syllables features (see §2). We first extracted a set of the closest nearest neighbours (100 closest syllables for each 5000 sampled syllable) and calculated the median distance, which is a simple estimate of how similar syllable features were at regions of high density (close to the centre of clusters). We did this for each bird every 5 days during development. Figure 4*b* shows the mean nearest neighbour distance for each group (error bars represent SEM) over development. As shown, the distances decreased with age in all three groups, indicating the emergence of clusters, but it decreased much more rapidly in WT and ST birds. Between day 50 and 65, the changes were particularly large whereas in the ISO group, changes began later, mostly after day 60, and occurred more slowly and gradually. In all three groups, the formation and tightening of clusters slowed down and eventually reached an asymptote, but this happened with a delay of about 10 days in the ISO group (around day 75–80). The time of rapid cluster emergence in ST and WT birds coincides with the time when major vocal changes typically occur to the phonetic structure of song syllables during normal song development.

In order to judge the effect of training, we used mixed effects linear regression (details in §2). We found a significant effect of training condition over development, with significant differences between ISO and WT birds (*p* < 0.01) and between ISO and ST birds (*p* < 0.01), but no difference between ST and WT birds (*p* = 0.76). There was no significant interaction between training condition and age, although it approached significance for the ISO and WT comparison (*p* = 0.09). This finding reflects the fact that developmental trajectories in different groups followed a similarly shaped curve, and the differences were stable over the course of development.

We next performed a posteriori tests to assess the differences between the groups separately on each day. The difference between the ISO and the ST birds was significant (*p* < 0.05) or approached significance from day 60 (with the exception of two days: 65 and 95). By contrast, there were no developmental time points when ST birds were significantly different from WT birds. This suggests that self-training induced the early emergence of syllable categories, just as training with an adult song model does in WT birds. The ISO birds, on the other hand, were invariably either significantly (*p* < 0.05) or marginally (*p* < 0.08) different from the other groups, and did not catch up with age.

**Figure 5.**
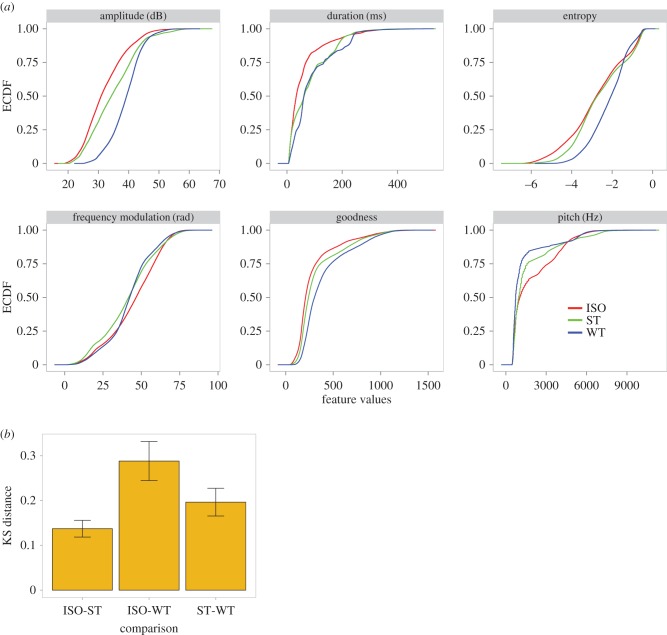
Distribution of ISO, ST and WT song features. (*a*) Song features of ST songs (green lines) are more WT-like (blue lines) than ISO song features (red lines): their cumulative distributions lie in-between the WT and the ISO distributions. (*b*) KS distance for combined acoustic features for the three possible comparisons between ISO, ST and WT songs.

Finally, we tested whether the clusters that ST birds generated were acoustically similar to song syllables of ISOs or to song syllables of WT birds. At the endpoint of song development, we compared the distribution of features across our three experimental groups (ST, ISO and WT). Figure 5 shows the ECDFs for six acoustic features. As shown, for most of the song features, the ST curve (green) lies in-between the ISO (red) and the WT (blue) curves. Figure 5*b* shows the combined Kolmogorov-Smirnov (KS) distances for all six song features for all three comparisons. The highest distance was between ISO and WT features and the lowest was between ISO and ST features. This confirms the visual impression that the songs of ST birds were indeed more WT-like than their ISO brothers', although ST song features were still slightly closer to ISO features than WT features.

## 4. Discussion

We found that providing birds with playbacks of their own delayed song throughout song development results in the early emergence of syllable types following a time-course similar to that observed in normal WT song development. ST birds developed phonetic categories despite the fact that their auditory input was initially a continuous and highly variable signal. Through self-feedback, acoustically continuous vocalizations evolved into categorical syllable types even in the absence of categorical input. The unexpected aspect of our finding is that providing the young birds with feedback of their own developing songs was sufficient to jumpstart a normal development process, which is typically deficient and delayed in ISO birds. Figure 4 suggests that cluster differentiation in all of the experimental groups reached an asymptote between days 70–85, at which time ISOs had not reached the level of category formation exhibited by ST birds. However, if the rate of change in ISO birds merely slowed down, there is a possibility that they would have eventually caught up with the ST birds, had they not run out of time by reaching the age of song crystallization. Although this is not likely (because the closing of the sensitive period in ISO birds is delayed [46,47] and ISOs are able to modify their songs beyond day 90), we cannot totally rule out the possibility that ISO song syllables would have developed further, as we discontinued song recording at day 120.

Self-tutoring had a lesser effect on the development of the phonetic structure of song notes (figure 5). A shift did occur towards WT feature distributions, but several acoustic features in the final ST songs (e.g. amplitude, entropy, goodness of pitch) remained more similar to the ISO siblings' song features than to those produced by WT birds. This could be due to genetic constraints on the phonetic components of songs. Indeed, certain vocal features (such as amplitude and frequency modulation) have been shown to be under strong genetic influence [48] and may reflect anatomical differences such as those in body size.

The final songs of the ST birds were highly diverse across birds: some ST songs were much more WT-like than others in terms of acoustic and syntactic structure. ISO zebra finch songs in general also show high variation both within and across individuals [29,49], presumably due to the lack of cultural selection that constrains WT song [50,51]. Inter-individual variation is also present in WT song, where it has been linked to anatomical differences in premotor song nuclei [52,53]. Moreover, juvenile birds exhibit variation in the particular learning strategy they employ in song imitation: some begin by singing the entire tutor song and modify the phonetic details within the temporal frame, while others begin with a serial repetition of one syllable type and then add in song syllables one by one [54]. The choice of the particular learning strategy is socially mediated but it is unclear how it relates to the final outcome of song learning. In a recent study, Okubo *et al.* showed that syllable differentiation, regardless of developmental strategy, occurs by the gradual splitting of neuron sequences in the premotor cortical area high vocal centre (HVC) [55]. Interestingly, the HVC shows strong auditory responses to song stimulation [56], but is temporarily unresponsive to song playbacks while the bird is singing [39]. Because in ISOs the only song input is the auditory feedback of the bird's own song, which is simultaneous with singing, the HVC cannot register it. Song learning in zebra finches requires HVC activation while listening to tutored song [38]. Our results support the notion that activation of the premotor song system during early development is necessary for initiating neuronal sequence splitting [55] that consequently leads to the formation of syllable categories.

Our findings suggest that the early emergence of categories is internally driven, rather than a simple reflection of imitating a categorical input. Consequently, song imitation can be seen as modulation, rather than the cause of the transition from continuous to categorical signals. In the absence of song feedback, categories evolved late and to a lesser extent, as indicated by the more diffuse syllable clusters observed in ISOs. Therefore, the emergence of categorical signals is strongly facilitated by presenting birds with song input, but the statistical structure of that early input, even if highly variable and continuous, appears to have little bearing on the differentiation of phonetic categories. The self-generation of distinct syllable types has implications at the level of song culture. Categorical signals should facilitate cultural transmission as they may be easier to learn and therefore more likely to remain stable. The strong effect of song input availability on cluster formation, which we observed in the ST birds, suggests that categorical formation is a socially mediated process. There is some tension between this socially triggered compression and simplification of vocal communication signals and sexual selection, because females show preference for males that produce more elaborate courtship signals, such as complex songs and large repertoires [57–60]. These opposing forces of simplicity to aid learning and complexity for sexual selection have a great impact on the evolution of song culture. This process is evidenced by the complex song of the domesticated Bengalese finch (*Lonchura striata* var. *domestica*) in contrast with the simple song of its wild ancestor, the white-rumped munia (*Lonchura striata*) [61]. Our study yields insights into which aspects of vocal development are determined by an innate developmental programme and which depend on external stimuli, and on the relationship between innate and culturally determined aspects of vocal culture. To our knowledge, the possible link between the extent of developmental compression and the stability of song culture had not previously been studied.

Our results also speak to the relationship between learning biases and song structure, and may have implications for human learning biases and linguistic structure. In a previous study, we showed that biased imitation of ISO song leads to WT song features over repeated episodes of transmission across generations [30]. In that study, the first bird in each chain of transmission was exposed to ISO song, and although the young birds readily imitated their ISO tutors, changes accumulated across generations, which resulted in WT song features within a few generations. That process is similar to what is observed in iterated learning experiments involving artificial language learning in humans, which have shown that linguistic structure evolves out of unstructured input across learners. Human studies have shown that over repeated episodes of iterated learning, languages become more learnable and compositional [22], more regular [62,63] and that categorical semantic structure emerges [64]; that is, linguistic structure evolves as a result of iterated learning. Collectively, these studies suggest that biases reflected in linguistic structure are amplified by transmission, and that this process requires transmission across multiple individuals. Our current study suggests relaxing this requirement: multiple individuals may not be necessary for learning biases to shape vocal output (as demonstrated by e.g. [41] for category learning) because iterations within a single individual in a closed sensory-motor loop may facilitate behavioural changes over development. Whether iterated self-training is as effective as inter-individual iterated learning in driving structural changes in vocal output remains an open empirical question. If a similar self-tuning of vocal behaviour towards universally observed linguistic features holds for humans, it could have important theoretical and practical implications.

The self-training approach that we have presented in this study may present a useful tool to investigate the parallels and differences between animal and human statistical learning abilities, and how they are employed during vocal learning. More specifically, it can be used to study how distributional information in the acoustic input guides vocal development. Future studies could investigate the extent to which normal development of phonetic categories (both in terms of production and perception) may be contingent on categorical input, and how similar the learning processes are in birds and humans. More generally, one may ask how the development of phonetic categories depends on the statistics of vocal input which, in songbirds, can be easily manipulated over developmental time-scales.

## Data accessibility

Example songs for the experimental birds and the spreadsheet containing the data used for feature analysis will be posted on the University of Edinburgh DataShare website; http://datashare.is.ed.ac.uk. The entire developmental dataset is stored on external HDs and samples will be provided upon request to O.F.
